# Obesity and caries in four-to-six year old English children: a cross-sectional study

**DOI:** 10.1186/s12889-018-5156-8

**Published:** 2018-02-17

**Authors:** Martha Paisi, Elizabeth Kay, Irene Kaimi, Robert Witton, Robert Nelder, Ruth Potterton, Debra Lapthorne

**Affiliations:** 1 Peninsula Dental School, Plymouth University, Plymouth, PL4 8AA UK; 2 School of Computing, Electronics and Mathematics, Plymouth University, Plymouth, PL4 8AA UK; 3 Office of the Director of Public Health, Plymouth City Council, Plymouth, PL6 5UF UK; 4 Public Health England, Follaton House, Road, Totnes, Plymouth, Devon TQ9 5NE UK

**Keywords:** Obesity, Caries, Children, Inequalities, Socioeconomic status

## Abstract

**Background:**

Obesity and caries are common conditions in childhood and can have significant implications on children’s wellbeing. Evidence into their association remains conflicting. Furthermore, studies examining the ssociation between obesity and caries commonly focus on individual-level determinants. The present study aimed to examine the association between obesity and caries in young English children and to determine the impact of deprivation and area-level characteristics on the distribution of the two conditions.

**Methods:**

This was a cross-sectional study among children in Plymouth city aged four-to-six years. Anthropometric measurements included weight and height (converted to Body Mass Index centiles and z-scores), and waist circumference. Caries was assessed by using the sum of the number of teeth that were decayed, missing or filled. A questionnaire was used to obtain information on children’s demographic characteristics, oral hygiene, and dietary habits. The impact of deprivation on anthropometric variables and caries was determined using Linear and Poisson regression models, respectively. Multiple logistic regression was used to assess the association between different anthropometric measures and caries. Logistic regression models were also used to examine the impact of several demographic characteristics and health behaviours on the presence of obesity and caries.

**Results:**

The total sample included 347 children aged 5.10 ± 0.31 (mean ± SD). Deprivation had a significant impact on caries and BMI z-scores (*p* < 0.05). Neither BMI- nor waist circumference z-scores were shown to be significantly associated with dental caries. Among the neighbourhood characteristics examined, the percentage of people dependent on benefits was found to have a significant impact on caries rates (p < 0.05). Household’s total annual income was inversely related to caries risk and parental educational level affected children’s tooth brushing frequency.

**Conclusions:**

No associations between any measure of obesity and caries were found. However, deprivation affected both obesity and caries, thus highlighting the need to prioritise disadvantaged children in future prevention programmes.

## Background

Obesity and dental caries (decay) are important health issues affecting a significant number of children worldwide [1–3]. Both have a negative impact on children’s health and cause a substantial financial burden to the healthcare system [1–3]. Studies show consistent social inequalities in the distribution of the two conditions, with disadvantaged children being affected to a greater extent by the two conditions [4, 5]. The presence of social gradient in health, where an inverse graded relationship between social status and health exists, has also been observed [6].

In England, over a fifth of five-year-olds are suffering from tooth decay or excess weight [7, 8]. Caries is also the most common reason why five to nine-year-old children, are admitted to hospital for tooth extractions [9]. Over a nine-year period (1997–2006), a 66% increase in tooth extractions due to caries was observed with five-year-old children being most commonly affected [10]. Evidence also shows that the presence of these two conditions in childhood is predictive of the two conditions later in life [11, 12].

A number of primary studies and systematic reviews have examined the association between obesity and caries as they share common risk factors such as diet. However, their results have been conflicting and equivocal [5, 13–18]. One study examining the association between obesity and caries in 12-year old children found that central (as indicated by waist circumference) but not general obesity (i.e. BMI) was positively associated with dental caries [19]. This raises the hypothesis that the association between the two conditions may be affected by the measure of obesity used.

Interestingly the vast majority of primary studies that have examined the association between obesity and caries have only examined the impact of individual-level factors (e.g. oral health habits, diet) on the association between the two conditions. However, evidence shows that the conditions in which people live, grow, and age and the broader environment, which also includes area-level characteristics, can have a significant impact on both conditions [1, 4]. The ‘Social Determinants of Health’ Framework emphasises that current inequalities in health are due to the daily living conditions and broader environment, which in turn influence the decisions and choices people make [20]. To the best of our knowledge, no study examining the association between the two conditions has to date examined the impact of area-level characteristics on the two conditions simultaneously nor has a study in the United Kingdom (UK) examined the association between different measures of obesity and caries risk.

### Aim

The present study aimed to investigate the association between different measures of obesity and caries in Plymouth children, UK. It also aimed to examine the impact of deprivation, individual behaviours and attributes as well as area-level characteristics on the distribution of the two conditions.

## Methods

### Plymouth geography

Plymouth is a relatively deprived city with non-fluoridated water in the South West of England (Devon) and has a population of 262,700 [21] residing in 39 neighbourhoods. The city’s economy can be characterised as low waged, with low rates of productivity [21]. Some of the neighbourhoods in the city are among some of the most deprived in the UK and based on household income, 1 in 5 Plymouth children are living in poverty [22].

### Study design and methodology

This was a cross-sectional study, and the sampling unit was all state-funded (known as ‘public’ in other countries) infant and primary schools in Plymouth. The six Plymouth localities, which are aggregations of adjacent neighbourhoods, were used as the strata. Sample size calculation was based on an odds ratio of 2.5 for the presence of caries in relation to high BMI that has been previously shown among 6-year-old children, [23] and the latest figures (when the sample size calculation was perfomed) concerning the percentage of Plymouth children who were obese (10% in 2013/14) and had dental decay (29.1% in 2009) [24, 25]. It was estimated that 20 schools would need to be invited in order to meet the target number of 352 children which was calculated to give 80% statistical power at a 5% level of significance. Schools were randomly selected using the random generator. All children at the reception year of the selected state-funded schools (i.e. aged four to six years) were eligible for participation. Any difficulties in obtaining a measurement from a child (e.g. due to a medical condition) were noted on the data recording form.

The recruitment took place between November 2014 and January 2015, and the data collection was conducted between January 2015 and May 2015. Following the Principal’s Approval for the school to participate in the study, an information sheet and a consent form were sent to all parents of children at the reception year of infant and primary school in the selected schools. The parents were asked to return the signed consent form to the school and were given the study’s questionnaire which was returned on the examination day.

### Anthropometric assessments

Anthropometric measurements were taken by a trained and calibrated researcher using standardised procedures and included weight, height and waist circumference [26]. Weight was measured in kilogrammes to two decimal places with an 877 Seca electronic scale. Height was assessed to the nearest 0.001 metres using a Seca 217 stadiometer. Waist circumference which was used as an indicator of central obesity was measured with a bodymorph tape to the nearest centimetre. Children’s BMI was calculated with the established formula [BMI=(weight in Kilograms/(Height in Metres x Height in Metres)], and was used to define general obesity. Definition of children’s weight status category was based on the updated UK 1990 growth reference centiles [27]. The ‘LMS Growth’ software was used to assign children in the corresponding percentiles and z-scores [28, 29].

### Dental examination

The dental examination was undertaken by a trained examiner and included visual examination of the teeth that were decayed, missing or filled (restored) summed into a single score (dmft). The methods and criteria used were those recommended by the British Association for the Study of Community Dentistry (BASCD) and also used in the UK National Epidemiology Programme of five-year-old children [30]. The examiner used a standardised light source, disposable gloves and intra-oral disposable mouth mirrors. Another trained researcher was recording the data according to the BASCD criteria. Intra-examiner reliability was examined on a random sample of 10% of children. The intra-class correlation coefficient was 0.99.

A questionnaire that was pilot tested on 20 parents/guardians visiting Plymouth University’s paediatric dental clinic, was administered to the parents/guardians and was used to obtain information on the following: a) children’s demographic and socioeconomic characteristics (i.e. parent’s/guardian’s age group, their level of education and annual household total); b) children’s oral health habits (i.e. tooth brushing frequency; child’s age when tooth brushing commenced and adult supervision during tooth brushing; child’s last dental visit) and c) the frequency of snacking of certain sugary foods/beverages in between meals.

Area deprivation was determined by the English Index of Multiple Deprivation (IMD) 2010, which is a relative indicator of deprivation at Lower Super Output Area (LSOA) level across England (LSOAs have an average population of 1500 people). The IMD is based on the principle that deprivation does not relate only to poverty [31]. Information on ‘income, employment, health, education, crime, access to services and living environment’ is used [20] to produce a score for every LSOA in England.

Several area-level characteristics previously shown to associate with the prevalence of the two conditions or with health-related behaviours were also examined in relation to the distribution of the two conditions. These included: the percentage of people on benefits; crime rates; density of fast food outlets; density of grocery shops and the presence of dental clinics. All were examined at neighbourhood level and in all cases values were corrected for the population size in each area.

### Statistical analyses

Children with incomplete data on anthropometric or dental assessments were excluded from the analyses. Listwise deletion was applied for missing information in the questionnaires. Continuous and categorical variables are presented as means (SD) and frequencies (%), respectively. At individual level, Linear regression models were used to examine the association between area deprivation (IMD 2010-continous independent variable) and anthropometric measures (outcome continuous variables: weight height, BMI, and waist circumference). The association between deprivation (IMD 2010-continous independent variable) and caries (outcome variable: dmft counts) was examined using Poisson regression models. The association between anthropometric variables (weight, height, BMI, waist circumference as independent continuous variables) and dental caries presence as indicated by dmft (dependent variable, binary response: yes or no), was examined using multiple logistic regression.

Generalised linear models (log-linear Poisson models) were used to investigate the impact of neighbourhood characteristics on obesity and caries rates. The initial model included all available covariates presented previously. Then starting with the least significant covariate (based on *p*-value) and using backwards elimination, the covariates were removed from the model one-by-one. Each time a covariate was removed, the model was refitted. This process was repeated until only significant covariates were left in the model (all with *p*-values> 0.05).

The impact of several demographic variables and health behaviours on the likelihood of overweight/obesity (binary response variable: yes or no) and caries (binary response variable: yes or no) was examined using logistic regression models. In these models, covariates were inserted one by one using stepwise regression (i.e. combining backward elimination and forward selection, allowing for covariates removed early in the process of backwards elimination to be reconsidered at a later stage).

Demographic factors (independent variables) included in the model were the parent’s age group (< 30 years –reference level- vs > 30 years) and education (up to secondary school–reference level-, Technical/College, University), the family’s total income (up to £25.599–reference level-, 26.000–36.399, 36.400 and above), and the gender of the child (reference level: female). Individual behaviours examined included were tooth brushing habits (once or less per day –reference level- vs ≥2 times per day), age when the child started having his/her teeth cleaned (< 1 year –reference level- vs > 1 year), presence of adult with children when teeth are being brushed (yes –reference level- vs no) and frequency of in-between meals food/drink consumption (using never as a reference). Logistic regression models with response ‘the presence/absence of dental decay’ and ‘being/not being overweight/obese’ (outcome variables) were fitted.

Differences in tooth brushing frequency (once or less per day –reference level- vs ≥2 times per day) were examined using a logistic regression model with IMD categories (IMD-1 most deprived–reference level-, 2, 3 least deprived), income (up to £25.599–reference level-, 26.000–36.399, 36.400 and above) and education (up to secondary school–reference level-, Technical/College, University) included as covariates in the model. Logistic regression was used to test differences in the response rate (for the questionnaires) between parents/guardians living in areas with different levels of deprivation (IMD 1–reference level-, 2 and 3).

The Statistical Package for the Social Sciences (SPPS, version 32) and the R software were used for the analyses. A *p*-value of less than 0.05 was considered to suggest statistical significance. The STROBE checklist has been used to report this study.

The present study has been approved by the Faculty of Health and Human Sciences Research Ethics Committee of Plymouth University (ref: 13/14–240).

## Results

Fourteen out of the 20 schools (70%) agreed to participate. The parents/guardians of 378 children agreed to their child’s participation in the study. Questionnaires were returned for 279 out of 378 children (74%). On the day of the examination, 19 children were absent from school, and another four had left the school. In addition, one child refused to have a dental examination and six did not want some of their anthropometric measurements to be taken. Thus, results for both anthropometry and oral health were available for 349 children and the results discussed below are based on this sample.

### Descriptive characteristics

The final sample included 349 children aged 5.10 ± 0.31 (mean ± SD) with 50.1% (*N* = 175) boys and 49.9% (*N* = 174) girls. The characteristics of the study sample are presented in Table 1.

**Table 1:** Sample characteristics and outcomes

	Mean ± SD
Age (years)	5.10 ± 0.31
	N (%)
Gender	
Boys	175 (50.1)
Girls	174 (49.9)
Ethnicity	
White	323 (92.6)
Mixed	9 (2.6)
Asian or Asian British	5 (1.4)
Chinese	1 (0.3)
Any other ethnic group	3 (0.9)
Unknown	5 (1.4)
Weight status	
Underweight	2 (0.6)
Healthy weight	279 (79.9)
Overweight	38 (10.9)
Obese	30 (8.6)
Dental caries	
Free of caries	239 (68.3)
Presence of caries (dmft> 0)	110 (31.7)
	Mean ± SD (95% CI)
dmft in total sample	1.01 ± 2.07 (0.79–1.23)
dmft among those with presence of caries	3.18 ± 2.57 (2.70–3.67)

The majority of children in the sample were of white background (*N* = 323, 92.6%). Most children (*N* = 279, 79.9%) were found to be in the healthy weight category (BMI: >2nd - <85th centile), while 19.5% (*N* = 68) were found to have excess weight (either overweight or obese: BMI: ≥85th centile). At national level, 21.9% of children in 2014/15 were either overweight or obese [32]. Around one-third of our sample (*N* = 107, 30.7%) were shown to live in the most deprived areas of Plymouth.

The children in the sample had on average 1.01 ± 2.07 (0.79–1.23) (mean dmft ±SD and 95% CI) teeth affected by caries which is higher than the national average of 0.8 at the same age group of children [7]. The mean number of teeth that were decayed among the participants was 0.86 ± 1.82 (0.67–1.06) (mean dt, SD and 95% CI) and the mean number of missing teeth was 0.06 ± 0.47 (0.01–0.11) (mean mt, SD and 95% CI). An average of 0.08 ± 0.13 (0.04–0.13) (mean ft, SD and 95% CI) teeth in the total sample were found to have been restored.

One hundred and ten children (31.7%) were found to have caries experience as shown by a dmft score above zero (dmft> 0) which is higher than the corresponding national figure of 24.7% [7]. These children had on average 3.18 teeth affected by caries (3.18 ± 2.574) (2.70–3.67) (mean dmft ±SD and 95% CI). At national level, the average dmft in five year olds with decay experience was 3.4 [7].

No difference between boys and girls in any of the BMI categories (*p* = 0.726) or in terms of caries presence (dmft> 0) was found in our study (*p* = 0.818). Variations in dental caries status based on sociodemographic characteristics, BMI category, and oral health behaviours are presented in Table 2. Amongst children who were reported to brush their teeth once a day or less, mean dmft was 1.21 (SE = 0.339). Those that brushed their teeth twice a day or more had a mean dmft of 0.82 (SE = 0.124). Children who were supervised whilst tooth brushing had a mean dmft of 0.80 (0.113) compared to 1.95 (0.685) for those who were not.

**Table 2:** Variations in dental caries status by sociodemographic characteristics, BMI status and oral health behaviours

Variable	N (% of responders)	dmft Mean (SE)	95% CI
Gender			
Male	174 (50.1)	1.07 (0.170)	0.74–1.41
Female	173 (49.9)	0.94 (0.143)	0.66–1.23
Parents’/guardians’ educational attainment			
Up to secondary school	60 (23.7%)	0.82 (0.209)	0.40–1.24
Technical/College	99 (39.1%)	1.32 (0.243)	0.84–1.81
University	94 (37.2%)	0.50 (0.127)	0.25–0.75
Household total income			
Up to £25.599	94 (37.5%)	1.37 (0.243)	0.89–1.86
£26.000 to £36.399	45 (17.9%)	0.44 (0.202)	0.04–0.85
£36.400 and above	86 (34.3%)	0.40 (0.113)	0.19–0.60
Prefer not to answer	26 (10.4%)	1.69 (0.486)	0.69–2.69
Tooth brushing habits			
Once a day or less	48 (19%)	1.21 (0.339)	0.53–1.89
Twice a day or more	205 (81%)	0.82 (0.124)	0.58–1.06
Age when children started brushing their teeth			
Under 1 year	155 (61.85)	0.83 (0.144)	0.54–1.11
1–2 years	79 (31.5%)	1.10 (0.249)	0.60–1.60
2–3 years	11 (4.4%)	0.55 (0.247)	−0.01-1.10
Cannot remember	6 (2.4%)	1.00 (0.683)	−0.76-2.76
Adult with child when brushing their teeth			
Yes	232 (91.7%)	0.80 (0.113)	0.58–1.02
No	21 (8.3%)	1.95 (0.685)	0.52–3.38
IMD category			
Most deprived (IMD 1)	180 (53.7%)	1.29 (0.173)	0.95–1.63
Middle deprived (IMD 2)	54 (16.1%)	1.11 (0.298)	0.51–1.71
Least deprived (IMD 3)	101 (30.1%)	0.45 (0.114)	0.22–0.67
BMI category			
Healthy (>2nd - < 85th centile)	279 (80.4%)	0.99 (0.124)	0.74–1.23
Overweight (≥85th - < 95th centile)	38 (11%)	1.18 (0.347)	0.48–1.89
Obese (≥95th centile)	30 (8.6%)	0.97 (0.360)	0.23–1.70

The IMD 2010 had a significant impact on BMI z-scores (general obesity) (*p* = 0.016), but not on any of the other anthropometric indices i.e. a 10 unit increase in IMD 2010 would increase BMI by 0.1. The IMD 2010 was also found to be significantly associated with caries (*p* < 0.05) (Table 3) i.e. a 10 unit increase in a child’s IMD 2010 would increase odds of decay by 1.34 times.

**Table 3:** Impact of IMD 2010 (independent variable) on anthropometric indices and caries (outcome/dependent variables)

	Coefficient (95% CI)	*P*-value
Weight	0.005 (−0.01, 0.02)	0.591
Height	−0.02 (− 0.06, 0.01)	0.226
BMI	0.01 (0.002, 0.02)	0.016*
Waist	0.01 (− 0.01, 0.03)	0.300
Caries	0.03 (0.02, 0.05)	< 0.001***

*P* value: Linear regression model for the impact of IMD 2010 on anthropometric variables and Poisson regression model for the impact of IMD 2010 on caries

*0.01 < *p* value < 0.05

***p value < 0.001

Results also indicated that none of the anthropometric measures (weight, height, BMI, waist circumference) were significantly associated with the presence of caries (dmft> 0) (Table 4).

**Table 4:** Association between anthropometric measures (independent variables) and dental caries status (dmft > 0 or dmft = 0) (outcome/dependent variable)

Anthropometric variables	OR (95% CI)	*P*-value
Weight	1.04 (0.96, 1.13)	0.301
Height	1.00 (0.96, 1.05)	0.881
BMI	1.14 (0.96, 1.34)	0.130
Waist circumference	1.02 (0.95, 1.10)	0.602

*P* value: Multiple logistic regression model

With regard to the questionnaire, on average, 254 responses were available for each question. The completed questionnaires that were returned were most commonly completed by the mother of the child (*N* = 240, 93%). A logistic regression model fitted to the data indicated that the parents/guardians of children living in the least deprived areas of Plymouth were 16% more likely to respond to the questionnaire than those living in the most deprived areas (*p* = 0.027) (data not presented in tables). It was also shown that higher household annual total income, was associated with lower probability of caries in children (*p* < 0.05). The odds of caries was 0.32 and 0.37 for income categories £26.000 to £36.399, and £36.400 and above, respectively, compared to the lowest income category (up to £25.599). Gender, tooth-brushing habits, age when children started brushing their teeth and the presence of adult when children brushed their teeth, did not significantly affect the odds of caries. These results are summarised in Table 5.

**Table 5:** Impact of demographic and health behaviour variables (independent variables) on caries (dmft > 0 or dmft = 0) (outcome/dependent variable)

Variable	N (% of responders)	Odds of caries ratio (category to reference level)	*P* value
Gender			
Female	173 (49.9)	Reference level	–
Male	174 (50.1)	0.94 (0.59, 1.48)	0.786
Parents’/guardians’ educational attainment			
Up to secondary school	60 (23.7%)	Reference level	–
Technical/College	99 (39.1%)	1.81 (0.88, 3.84)	0.114
University	94 (37.2%)	0.93 (0.43, 2.06)	0.862
Household total income			
Up to £25.599	94 (37.5%)	Reference level	–
£26.000 to £36.399	45 (17.9%)	0.32 (0.13, 0.74)	0.011
£36.400 and above	86 (34.3%)	0.37 (0.18, 0.73)	0.005
Prefer not to answer	26 (10.4%)	1.27 (0.52, 3.07)	0.588
Tooth brushing habit			
Once a day or less	48 (19%)	Reference level	–
Twice a day or more	205 (81%)	0.84 (0.43, 1.71)	0.623
Age when children started brushing their teeth			
Under 1 year	155 (61.85)	Reference level	–
1–2 years	79 (31.5%)	1.19 (0.65, 2.16)	0.564
2–3 years	11 (4.4%)	1.27 (0.26, 5.03)	0.745
Cannot remember	6 (2.4%)	1.27 (0.17, 6.74)	0.789
Adult with child when brushing their teeth			
Yes	232 (91.7%)	Reference level	–
No	21 (8.3%)	2.54 (1.00, 6.48)	0.052

*P* value: Logistic regression model

Table 6 shows that none of gender, parents’/guardians’ educational attainment and household total income affected the odds of overweight/obesity.

**Table 6:** Impact of demographic and health behaviour variables (independent variables) on overweight/obesity (yes/no) (outcome/dependent variable)

Variable	N (% of responders)	Odds of overweight/obesity ratio (category to reference level)	*P* value
Gender			
Female	173 (49.9)	Reference level	–
Male	174 (50.1)	1.11 (0.65, 1.90)	0.702
Parents’/guardians’ educational attainment			
Up to secondary school	60 (23.7%)	Reference level	–
Technical/College	99 (39.1%)	0.91 (0.40, 2.15)	0.828
University	94 (37.2%)	1.28 (0.57, 2.97)	0.559
Household total income			
Up to £25.599	94 (37.5%)	Reference level	–
£26.000 to £36.399	45 (17.9%)	0.46 (0.16, 1.17)	0.122
£36.400 and above	86 (34.3%)	0.75 (0.36, 1.54)	0.436
Prefer not to answer	26 (10.4%)	0.90 (0.30, 2.41)	0.841

*P* value: Logistic regression model

Our results also indicated that the reported frequency of tooth brushing was not affected by IMD category (*p* > 0.05) nor the household total income (*p* > 0.05). However, children with University-level educated parents were 71.6% more likely to brush their teeth two or more times daily compared to children with secondary school-level educated parents (*p* < 0.05). The above data are not presented in tables.

Regarding covariates at neighbourhood level, albeit the effect size was small, there is evidence that the percentage of people on benefits affected caries rates (*p* = 0.002). Namely, a 1% increase in the percentage of people on state benefits would increase the caries rate by 1.04 times. None of the neighborhood level covariates had an impact on obesity (*p* > 0.05).

With regard to individual health behaviours, none of the reported covariates was associated with being overweight/obese. However, the presence or absence of dental decay appeared to be associated with the reported consumption of sugar confectionery, cakes and pastries, yoghurt desserts and fruits (fresh and dried) (*p* < 0.05).

Given that children from all localities were represented in the sample population and that a random selection of schools was conducted (where all children at reception class were eligible for participation), we believe that the sample population is representative of Plymouth children in this age group. Analysis of data from the National Child Measurement Programme in Plymouth [32], which measures the weight and height of children aged four to five years in almost all state-funded infant and primary schools in the city, show a similar distribution of ethnic background and gender in the city as that of our sample. In addition, distribution of children in IMD categories (1 to 5) in our sample is similar to that of NCMP [32], which further supports the generalisability of our findings to the Plymouth four to six year old population.

## Discussion

In the present study, no association between obesity and caries was found. This is in agreement with previous studies which used BMI as an indicator of weight status and which showed that there was no difference in caries prevalence or severity based on BMI status [18, 33]. It was initially hypothesised that BMI may not be the most suitable tool for assessing a child’s weight status when investigating the association between obesity and caries. This is because BMI cannot differentiate between fat mass and muscle or bone mass [34] and because in contrast to other indices of obesity (i.e. waist circumference) it does not indicate the location of fat [19]. In contrast, central obesity has been postulated to have the possibility of offering a physiological basis of an association between the two conditions, as the location of fat is associated with increased risk of disease [19, 35]. In another study, obesity was found to be related to decreased flow rate of saliva (predisposing factor to caries) as well as caries, and therefore it is possible that the negative impact of obesity on the flow rate of saliva may be regulated by inflammatory mediators which are commonly due to central obesity [36]. However, in the present study, the lack of association between obesity and caries remained when waist circumference rather than BMI was used to measure obesity. Therefore, the findings of our study, contrary to some other studies [5, 37] do not support the hypothesis that the association of obesity with caries may depend on the measure of obesity used. Further appropriately-powered studies examining the association between caries and different measures of obesity are therefore deemed necessary.

Given the progressive nature of obesity and caries, it is possible that an association between the two does not manifest until later in life. Thus, the young age of the participants in this study may have contributed to the lack of an observed association between the two conditions. This hypothesis is supported by several reports which suggest that the association between obesity and caries is more profound in the permanent dentition and thus is only seen in older children [13]. Thus, due to their young age, the duration that the participants in our sample had suffered from excess weight or were exposed to the cariogenic environment may have not been adequate to impact on the association between the two conditions. Another reason that obesity and caries may become associated in older children could be the reduction in parental control over eating and oral health habits, and the increased sedentary lifestyles and unhealthy dietary habits more commonly seen in older children than younger ones (i.e. higher frequency of fatty and sugary snacks) [13]. Future longitudinal cohort studies examining the association between the two conditions at different life stages would be particularly useful.

The current study found that deprivation is associated with children’s obesity status. This is in agreement with many studies which show that children in deprived areas are more likely to be obese than their peers in less deprived areas [32, 38]. In terms of individual measures of socioeconomic status (SES), the dietary habits of disadvantaged children and therefore their vulnerability to weight gain have been shown to be affected by household income and food cost [39, 40]. Lack of money means that parents opt to purchase cheaper food for their children which tend to be higher in fat and sugar than the more expensive ones [41, 42]. However, in the present study household income was not related to weight status, nor did the parents of obese children report higher frequency of sugar consumption than their healthy weight counterparts. The lack of a statistical association between consumption of sugary items and excess weight in our study may be a result of recording only the frequency of consumption and not the amount. Although frequency and amount of sugar consumption are highly related, the amount rather than frequency of sugar consumption is thought to be the most crucial factor for obesity [13], whilst the opposite is true for caries. Furthermore, the use of self-reported data could probably, at least, explain why our results did not show a sugary food/obesity association. Research has shown that parents frequently over report the intake of foods/drinks perceived as healthy and under report the intake of unhealthy items [43]. As underreporting of intake is more common among parents of children who are overweight/obese [44], the possibility that the use of self-reported data affected our results cannot be excluded.

The present study, similarly to earlier studies, identified a strong association between area deprivation and dental caries, meaning that children living in deprived areas suffered from caries severity to a greater extent [5, 19]. This finding provides supports to the Social Determinants of Health Theory, which highlights that everyday life conditions and the wider environment can affect the development of chronic conditions [20]. Furthermore, a household’s total income was inversely associated with a child’s dental caries status and parental educational level affected children’s tooth brushing frequency, with children having better-educated parents being significantly more likely to brush their teeth twice a day or more. Both household total income and parental educational level, which were used as measures of individual SES in the present study, have previously been shown to affect caries experience [5] and two systematic reviews found that both indicators were inversely associated with dental caries severity or prevalence [45, 46]. Although this cannot be substantiated by the present study, possible mechanisms by which family income and parental education could affect dental caries include cost of food (which may force people to purchase foods which are cheaper but high in sugar) [41] and lack of awareness of the importance of oral hygiene [47]. As delivery of fluoride by daily tooth brushing is considered to be the most effective caries preventive measure [48], the association between parental education and children’s tooth brushing frequency may be indicating one mechanism which explains how oral health inequalities are perpetuated across generations.

The percentage of people dependent on state benefits per neighbourhood was shown to be associated with increased caries rates, appearing to explain at least in part the distribution of caries in the city. This finding further reinforces the notion that the broader environment has an impact on our health [1, 6, 49]. WHO advocates that reduction in the inequalities in health, present both between and within countries, will be achieved only when conditions which create inequalities are targeted [20]. Watt and Sheiham [49] also highlight that in addition to targeting behavioural risk factors that are common to many conditions, targeting the underlying causes of chronic diseases (socioeconomic and political environment) is the most promising approach for tackling oral health inequalities. Given the cross-sectional analyses of the work, some caution must be applied, but the results strongly suggest that aspects of the lived-in environment impact on caries and this gives support to calls to tackle the condition from a holistic perspective.

Taking into account our results and the literature which clearly shows that interventions targeting only individual behaviours and lifestyles are largely ineffective in improving health conditions [1, 50], further studies should explore more deeply the socioeconomic influences on both obesity and caries and how these may influence individual choices. This can include the application of geographic systems to examine the impact of several area-level characteristics on the emerging patterns of the two conditions as well as their relationship at the geographical level. Such analysis can enable identification of vulnerable and high risk areas in need of intervention [51]. It can also help us to understand how broader health determinants such as aspects of the physical, political and socio-economic environment interact with individual characteristics and behaviours to produce the emerging patterns [52, 53]. This approach can also enable the broader common determinants of obesity and caries to be identified and targeted in a coordinated way.

### Limitations

The present study was limited by its cross-sectional design. However, in contrast to all previous studies, rather than focusing only on individual-level factors, we also examined the impact of neighbourhood-level characteristics on the two conditions.

A second potential limitation of the study is that the anthropometric variables are only indirect measures of an individual’s nutritional status [54]. Laboratory methods for assessing body composition (e.g. Dual-energy X-ray Absorptiometry-DXA) are more accurate, however, their high cost and difficulties in transportation limit their application in epidemiological studies. Finally, reporting biases in the questionnaire data may have influenced our findings whilst the study was not powered to take into account clustering of pupils in schools.

## Conclusions

The present study found no evidence of an association between obesity and caries in young Plymouth children, regardless of the indicator of obesity used. However, the finding that deprivation affects both conditions highlights the vulnerability of children living in deprived areas to both excess weight and caries. The importance of prioritising disadvantaged areas in future intervention strategies is therefore reinforced by this study, as well as the notion that the broader environment impacts the development of both conditions. Our findings finally suggest that the prevention of chronic lifestyle-related conditions may lie in the provision of supportive environments (in education, employment, and housing) rather than the solutions being only the ‘correction’ of unhealthy behaviours.

Future longitudinal studies exploring the exact nature of the association between obesity measures and caries in different age groups and the impact of area-level characteristics on individual behaviours are warranted.

## Abbreviations

BASCD: British Association for the Study of Community Dentistry
BMI: Body Mass Index
DXA: Dual-energy X-ray Absorptiometry
IMD: Index of Multiple Deprivation
LSOA: Lower Super Output Area
SPSS: Statistical Package for the Social Sciences
UK: United Kingdom
